# Prevalence and clinical significance of co-existing mutations in *MED12* and *FH* in uterine fibroids of Australian women

**DOI:** 10.3389/frph.2023.1081092

**Published:** 2023-04-11

**Authors:** M. Fairuz B. Jamaluddin, Prathima B. Nagendra, Yi-An Ko, Preety Bajwa, Rodney J. Scott, Pravin Nahar, Pradeep S. Tanwar

**Affiliations:** ^1^ Global Centre for Gynecological Diseases, University of Newcastle, Callaghan, NSW, Australia; ^2^ School of Biomedical Sciences and Pharmacy, University of Newcastle, Callaghan, NSW, Australia; ^3^ Division of Molecular Medicine, NSW Health Pathology, John Hunter Hospital, Newcastle, NSW, Australia; ^4^ School of Medicine and Public Health, University of Newcastle, Callaghan, NSW, Australia; ^5^ Department of Maternity and Gynaecology, John Hunter Hospital, New Lambton Heights, NSW, Australia; ^6^ Hunter Medical Research Institute, New Lambton Heights, NSW, Australia

**Keywords:** ANM, adjacent normal myometrium FH, fumarate hydratase MED12, mediator complex subunit 12, leiomyoma

## Abstract

Uterine fibroids are exceedingly common benign tumours of the female reproductive system and cause severe symptoms, including acute pain, bleeding, and infertility. Fibroids are frequently associated with genetic alterations affecting mediator complex subunit 12 (*MED12*), fumarate hydratase (*FH*), high mobility group AT-hook 2 (*HMGA2*) and collagen, type IV alpha 5 and alpha 6 (*COL4A5-COL4A6*). Recently, we reported *MED12* exon 2 mutations in 39 out of 65 uterine fibroids (60%) from 14 Australian patients. The aim of this study was to evaluate the status of *FH* mutations in *MED12* mutation-positive and mutation-negative uterine fibroids. *FH* mutation screening of altogether 65 uterine fibroids and corresponding adjacent normal myometrium (*n* = 14) was carried out by Sanger sequencing. Three out of 14 patients displayed somatic mutations in *FH* exon 1 in addition to harbouring *MED12* mutation in uterine fibroids. This study is the first to report that the mutations in *MED12* and *FH* co-exist in uterine fibroids of Australian women.

## Impact statement

**What is already known on this subject:** The majority of fibroids harbor mutations as high as 60% in relation to the most common genetic driver in uterine fibroid that is *MED12*.

**What the results of this study add:** Sanger sequencing has identified that three out of 14 patients displayed somatic mutations in *FH* exon 1 and these patients were also harboring *MED12* mutations in their fibroid.

**What the implications are of these findings for clinical practice and/or further research:** The events of both *MED12* and *FH* mutations co-occur in uterine fibroid sets the foundation for further functional studies to investigate the relationship between *MED12* and *FH* in leiomyogenesis. The impact of this study may guide the development of new approaches for managing millions of patients affected by this disease.

## Introduction

Uterine leiomyomas (also termed fibroids) are extremely common tumours of the female reproductive tract affecting as high as 70% of women by the age of 50 (1). The clinical symptoms attributable to fibroids include pelvic discomfort, profuse menstrual bleeding, reduced fertility, and pregnancy complications (2). Fibroids are the leading indication for hysterectomy among US women accounting for over 200,000 procedures performed annually (3). An improved understanding of the aetiology and pathogenesis that drive fibroid growth may guide the development of new treatment options for millions of patients who suffer from the symptoms of fibroids.

In recent years, significant progress has been made in understanding the genetic basis of uterine leiomyogenesis through whole genome sequencing studies (4, 5). There are currently four well-studied genetic alterations that are present in human uterine leiomyomas, namely, mediator complex subunit 12 (*MED12*) mutation, high mobility group AT-hook 2 (*HMGA2*) overexpression, biallelic fumarate hydratase (*FH*) inactivation and collagen, type IV alpha 5 (*COL4A5*) and collagen, type IV alpha 6 (*COL4A6*) deletion (5, 6). Mutations in the *MED12* gene are the most common and present in approximately 70% of patients (5).

A previous study has shown that *MED12* and *FH* aberrations are mutually exclusive, suggesting that they are independent genetic events that occur in the fibroid formation (7). *MED12* belongs to a highly conserved multiprotein complex Mediator that involves in the processing of RNA polymerase II transcription (8). Oncogenic anomalies in the N-terminal part of *MED12* are the most common and occur in nearly 70% of uterine fibroid (4). Fibroids may also harbour germline mutations of the *FH*, which encodes the tricarboxylic cycle enzyme fumarase, predisposing women to the hereditary leiomyomatosis and renal cell cancer (HLRCC) syndrome (9).

Recently, we identified 3% and 60% of uterine fibroids harbour specific mutations in *MED12* exon 1 and exon 2 respectively of 65 uterine fibroids studied that were collected from Australian women (10). The majority of the mutations were predominantly missense mutations affecting a codon encoding glycine 44 in exon 2, which is a known mutational hotspot in *MED12*. In this study, we examined the status of *FH* in Australian patients with *MED12* mutations and found three patients (3/14 patients) with co-existing *FH* and *MED12* mutations in the same fibroid. Histopathological examination was performed to determine the significance of these co-existing mutations on fibroid growth.

## Materials and methods

### Subjects

Sixty-five uterine fibroids of different sizes from 14 patients, and 14 ANM from the same patients (1 ANM per patient), were obtained following hysterectomy procedures at the John Hunter Hospital, Newcastle, NSW, Australia. The human tissue collection and experimentation were approved in accordance with the guidelines of the Institutional Human Research Ethics Committee at the University of Newcastle. The collected tissues were processed as described in (11). We categorized fibroids into three groups based on their tumour size: small (diameter <2 cm), medium (diameter 2 cm–4 cm), and large (diameter >4 cm) (10).

### Mutation screening

Small portion of fresh frozen fibroid or ANM tissues (approximately 25 mg) were used for genomic DNA extraction. Isolation of genomic DNA was performed using the QIAamp DNA Mini kit (Qiagen) according to the manufacturer's instructions. DNA from fibroid and ANM tissue samples were amplified with Immolase^™^ DNA polymerase (Bioline) using ten designated intronic pairs of PCR primers that target the 10 *FH* exons, as described previously (12). 5 µl of PCR product was run on 1.5% agarose gel (Bioline) to verify specificity to the reaction, and the rest of the products were purified using QIAquick PCR Purification Kit (Qiagen) according to the manufacturer's instructions. The purified PCR products were sequenced using the Sanger method, as previously described (10). Sequence chromatographs were analysed for somatic mutations in 10 *FH* exons using the Mutation Surveyor software (Softgenetics, State College, PA, USA).

### Histological and immunohistochemical (IHC) analysis

Collected fibroid or ANM tissues were fixed for 20 h in neutral buffered formalin, followed by embedding them into paraffin blocks. Six micron thick tissue sections were obtained. Tissue was cleared in xylene and rehydrated in increasing concentrations of ethanol and, finally, water. Haematoxylin and eosin staining was performed using the standard protocol. The IHC protocol followed is described by us in (11). Primary antibody, Ki67 (1:800, ab16667, Abcam, USA), and biotinylated secondary goat anti-rabbit (ready to use; Biogenex, CA, USA), were used. Stained slides were imaged at high resolution on the Aperio Scanscope slide scanner. The gain and exposure time were set constant across tissue samples. Analysis for intensity and the number of cells was done using the Halo Image analysis software (Indica labs, NM, USA).

### Statistics

All indicated values are in mean ± SEM and were subjected to unpaired Student's *t*-test to assess differences between *MED12* and *MED12 *+ *FH* groups with a *p*-value threshold set to less than 0.05 for statistical significance. All analyses were carried out on Graph Pad Prism 6.0.

## Results

### *MED12* and *FH* genetic alterations co-exist in uterine fibroids

To determine the status of *FH* in uterine fibroids, we performed Sanger sequencing and screened for *FH* mutations in 14 Australian patients. 13 out of 65 fibroids from 14 patients were positive for *FH* mutations (13/65; 20%; Table 1). In particular, we identified three out of these 14 patients harboured the *FH* mutation-positive (namely ULM4, ULM7, and ULM8). We have screened all these patients for *MED12* aberrations and identified that 60% fibroids (39/65) were positive for *MED12* mutations/deletions. The remaining 26 fibroids were found to be *MED12* mutation-negative (10).

**Table 1:** Summary of somatic *MED12* and *FH* mutations observed in fibroids of various sizes from three patients. Fibroids were classified as small (diameter <2 cm), medium (diameter 2 cm–4 cm), and large (diameter >4 cm) based on their size.

					*MED12* mutation status		*FH* status
Index	Patient	Age (years)	Sample ID	Description	Exon 1	Exon 2	Exon 1
1	1	56	ULM 4.1	Large fibroid	wt	c.115_135del, p.L39_F45del	c.147G > A, p.L28L
2	1	56	ULM 4.2	Fibroid large (2nd site)	wt	c.115_135del, p.L39_F45del	c.147G > A, p.L28L
3	1	56	ULM 4.3	Small fibroid within Large	wt	c.114_134del, p.L39_F45del	c.147G > A, p.L28L
4	1	56	ULM 4.4	Large fibroid intact	wt	c.115_135del, p.L39_F45del	c.147G > A, p.L28L
5	1	56	ULM 4.5	Adjacent normal myometrium	wt	wt	c.147G > A, p.L28L
6	1	56	ULM 4.6	Small fibroid	wt	c.114_134del, p.L39_F45del	c.147G > A, p.L28L
7	2	56	ULM 7.1	Small fibroid	wt	c.131G > T, p.G44V	wt
8	2	56	ULM 7.2	Very small fibroid	wt	c.131G > A, p.G44D	wt
9	2	56	ULM 7.3	Medium fibroid	wt	c.131G > A, p.G44D	wt
10	2	56	ULM 7.4	Medium II fibroid	wt	c.130G > C, p.G44R	wt
11	2	56	ULM 7.5	Very small fibroid	wt	wt	wt
12	2	56	ULM 7.5.1	Very small fibroid	wt	wt	wt
13	2	56	ULM 7.6	Large fibroid	wt	c.126_132del, p.K42_G44del	c.64A > C, p.M1L
14	2	56	ULM 7.7	Adjacent normal myometrium	wt	wt	wt
15	2	56	ULM 7.8	Large fibroid	wt	c.130G > A, p.G44S	wt
16	3	58	ULM 8.1	Adjacent normal myometrium	wt	wt	c.80G > C, p.R6P; c.86T > C, p.L8P; c.88G > C, p.A9P; c.106G > C, p.V15L
17	3	58	ULM 8.2	Large fibroid	wt	wt	wt
18	3	58	ULM 8.3	Small fibroid	wt	c.131G > A, p.G44D	c.80G > C, p.R6P
19	3	58	ULM 8.4	Smallest subserosal fibroid	wt	c.131G > A, p.G44D	wt
20	3	58	ULM 8.5	Large fibroid sub mucosal	wt	wt	c.88G > C, p.A9P
21	3	58	ULM 8.6	Normal adjacent to large fibroid	wt	wt	c.88G > C, p.A9P
22	3	58	ULM 8.7	Midsize fibroid	wt	wt	wt
23	3	58	ULM 8.8	Intral mural midsize fibroid	wt	wt	c.88G > C, p.A9P
24	3	58	ULM 8.9	Mid size intra mural fibroid	wt	wt	c.86T > C, p.L8P; c.88G > C, p.A9P; c.106G > C, p.V15L

**Figure 1:**
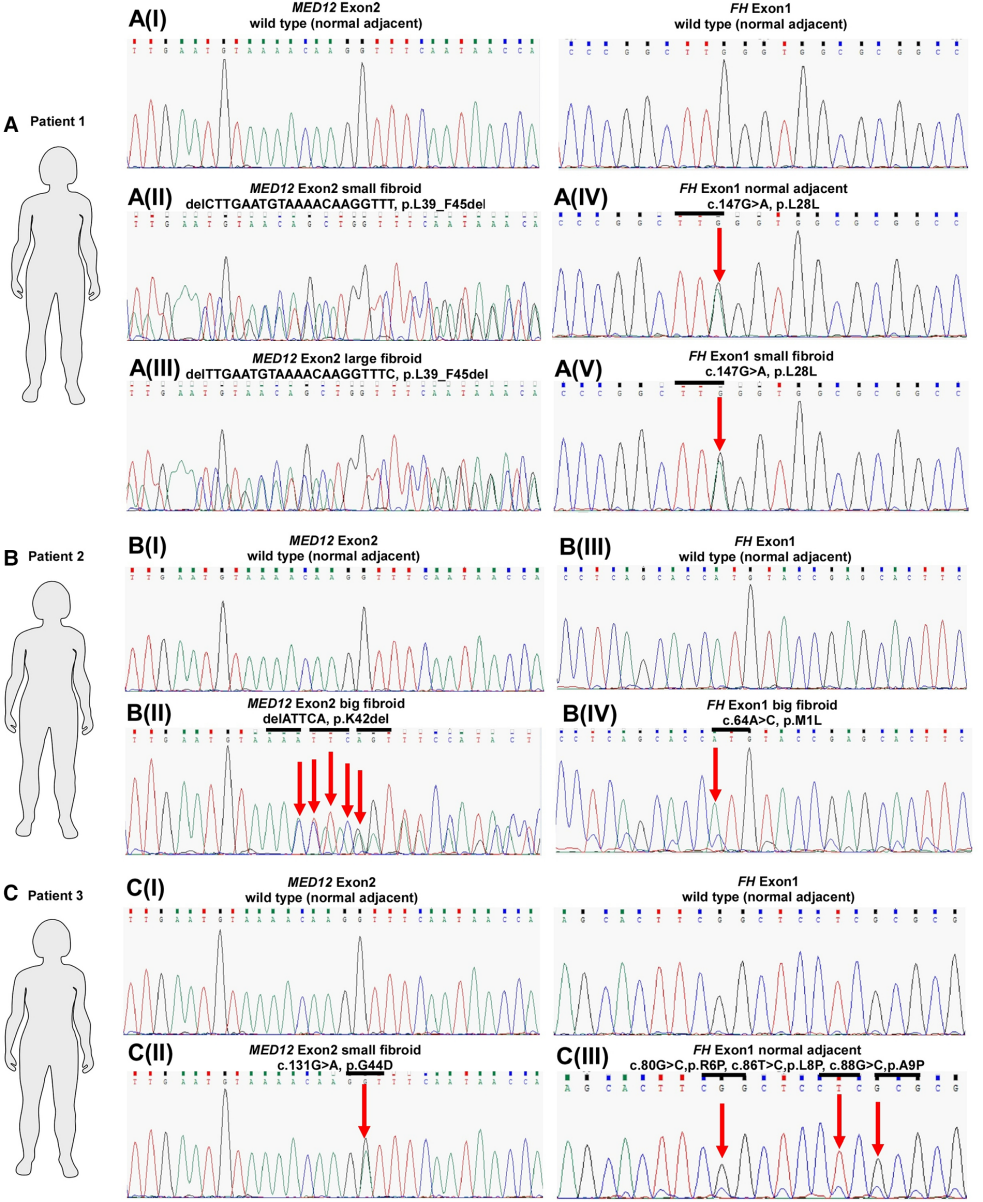
*MED12* and *FH* mutation status in three patients with uterine leiomyomas. Sequence chromatograms of selected fibroids and corresponding ANM samples in terms of *MED12* vs. *FH* mutation are shown. Codon in *MED12* and *FH* is highlighted by the horizontal bars above the traces. Arrows indicate mutated bases.

**Figure 2:**
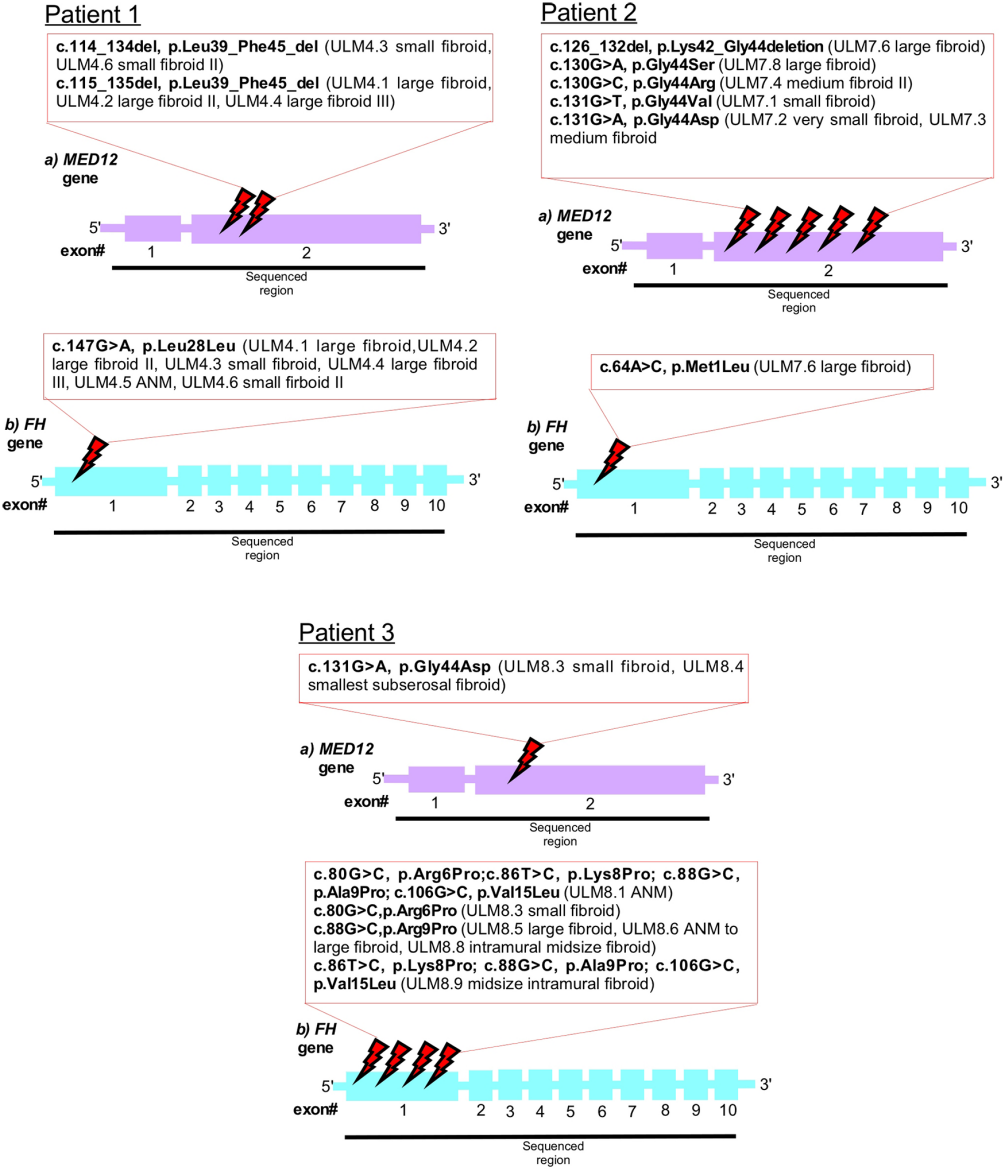
Schematic representation of the *MED12* (**A**) and *FH* (**B**) genes. Mutated bases are indicated in the red box. Intronic regions are not drawn.

Our genetic analysis highlighted that multiple fibroids and ANM within patient one (ULM4) displayed silent mutations, all affecting codon 28 in exon 1 of *FH* (Figures 1, 2 and Table 1). Patient one had five fibroids predominantly affected with a deleterious mutation (p.L39_F45del) in *MED12* exon 2. No *MED12* mutations were present in the patient's ANM tissue DNA (Table 1).

In Patient two, only one missense mutation was detected in codon M1L of *FH* exon 1 in the large fibroid (sample ULM7.6) (Figure 1 and Table 1). More specifically, this fibroid tissue displayed deletion mutation (p.K42_G44del) in *MED12* exon 2. Other anomalies in *MED12* exon 2 (including G44D, G44R, G44S and G44V) are also present in other fibroids but they are not affected by *FH* (Table 1 and Figure 2).

In Patient three, we observed multiple mutations affecting codon 6, 8, 9 and 15 in *FH* exon 1 and all of these mutations were also present in the patient's ANM (sample ULM8.1). Missense mutations were also found across various size tumours and these include R6P and A9P (Table 1 and Figure 2). Another hot spot was detected in codons L8P, A9P and V15L in medium size intra mural fibroid (sample ULM 8.9) (Table 1 and Figure 2). Interestingly, one (sample ULM8.3) out of these five tumours with *FH* mutations also had a missense mutation, affecting codon 44 in exon 2 of *MED12* (Figures 1, 2 and Table 1). We sought to analyse the mutation in exon 1 as well across the three patients, and no changes were observed. All of the mutations residing in *FH* exon 1 had not been previously reported. This analysis confirmed that mutations in *FH* and *MED12* co-exist for the first time in uterine fibroids in Australian women.

### Histopathological analysis of uterine fibroids with *MED12* and *FH* mutations

**Figure 3:**
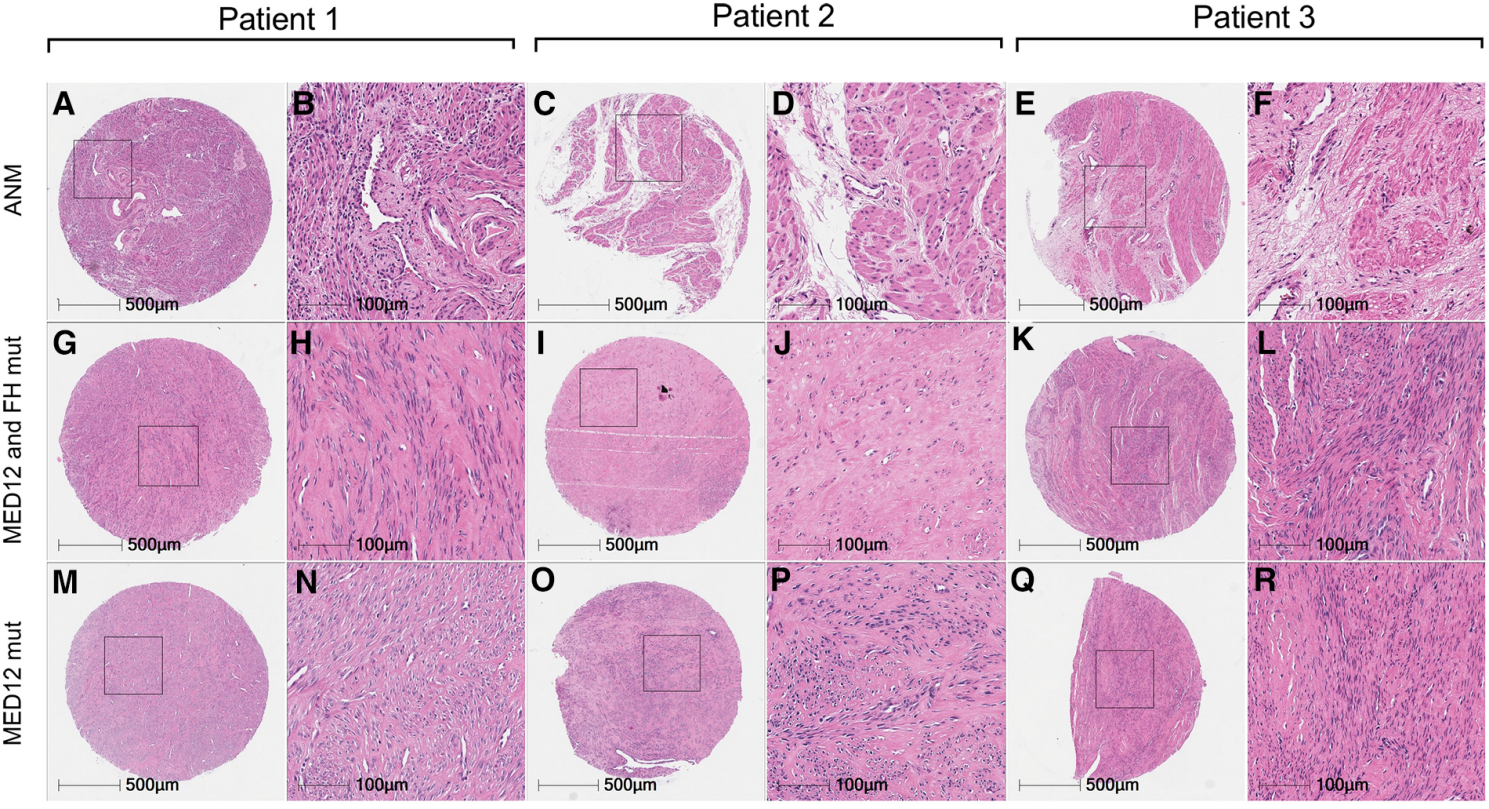
Comparative histology of uterine leiomyomas with paired adjacent normal myometrium. Haematoxylin and eosin staining of uterine leiomyomas from three patients with mutations in *MED12* only (**M–R**) vs. *MED12 + FH* (**G–L**) in comparison with adjacent normal myometrium (**A–E**) from the same patients.

**Figure 4:**
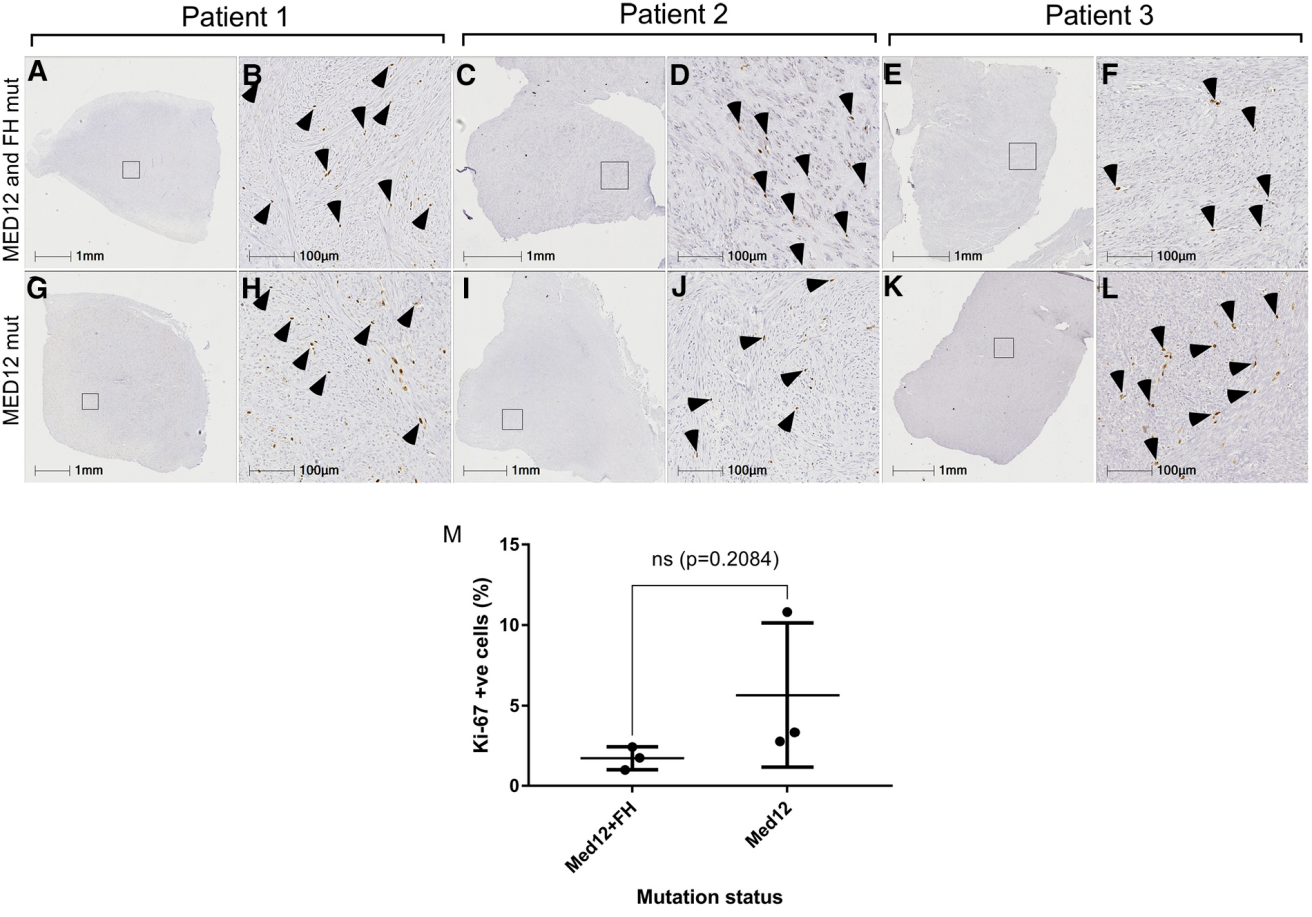
Mitotic index of *MED12* mutation vs. *MED12* and *FH* mutation leiomyomas. Ki-67 staining for *MED12* mutation (**G–L**) and *MED12* and *FH* mutation (**A–F**). Arrowheads indicate the Ki-67 positive cells. No significant difference was observed in the percentage of Ki-67 positive cells between these two groups (**M**).

Histological analysis of all fibroids and ANM was performed to reaffirm the clinical diagnosis in patients. All fibroids collected showed an atypical lack of organised muscle bundles, higher amounts of extracellular matrix deposition, compressed muscle fibers, and the presence of single cell type (smooth muscle cells), which are hallmarks of leiomyomas, compared to the defined vasculature and smooth muscle bundles in ANM (Figure 3). No significant differences in the number of smooth muscle cells and amount of extracellular matrix deposition were seen between *MED12* mutation-positive (Figures 3M–R) and *MED12 *+ *FH* mutation-positive (Figures 3M,G–L) fibroids. To understand if driver mutations in more than one gene would give a distinguishable growth advantage, the number of mitotic entities per 20X microscopic field was analysed for both groups by immunohistochemical staining for Ki-67 (Figure 4). The Ki-67+ve cells were capped at below 10% (characteristic of benign tumours). There was a slight increase in the number of mitotic entities per visible field, which was statistically insignificant (*p*-value = 0.2084).

## Discussion

Uterine fibroids are benign tumours that affect the reproductive health of millions of women (13–15) It is a main cause of infertility and hysterectomy (3, 15). The majority of the tumours (more than 70%) are identified by specific mutations in *MED12* exon 2 irrespective of ethnicity (16). On the other hand, there are other driver mutations, including *HMGA2*, *FH* and *COL4A5-COL4A6* (6). While these four gene alterations are shown to be mutually exclusive in some patients (17, 18), there have been other reports involving *HMGA1* or 7q aberrations of CUX1 gene that co-occur with *MED12* mutations (17). Holzmann and colleagues described a patient with *MED12* mutations and *HMGA12* rearrangements in her leiomyomas (19). Similarly, a recent study in the United States confirmed a patient carrying both driver mutations affecting *FH* and *MED12* in a single fibroid (20).

The aim of this study was to search for somatic mutations in *FH* gene in *MED12* mutation-positive and mutation-negative uterine fibroids of Australian women. Altogether 65 uterine fibroids and corresponding ANM tissues from 14 patients were successfully analysed using Sanger sequencing method. Three out of 14 patients displayed somatic mutations in *FH* exon 1 with co-existing mutations in *MED12*.

In Patient One, a deletion mutation L39_F45 in *MED12* exon 2 were detected in all of the fibroid tissues except the ANM (Table 1 and Figure 2). This agrees with previous studies that reported two mutation types in *MED12* exon 2 (including missense and inframe insertion-deletion mutations) identified in the human tumours (4, 16). Our genetic analysis investigation on *MED12* exon 2 mutations also found both. The effects of this mutation (L39_F45 deletion), which is located at the beginning of *MED12* region, are likely to impair protein-protein interactions between MED12 and Cdk8-Cdk19 and Cyclin C, which ultimately leads to the inhibition of the transcription. This statement was supported by previous expression profiling and functional studies that mutant MED12 protein showed a weak binding affinity with Cdk8 and Cyclin C compared to wild-type MED12 (19, 20). Apart from the deletion mutation that exists in this patient, we found another silent mutation affecting codon 28 in exon 1 of *FH* in this patient's normal tissue DNA. A similar change (L28L) in this region of *FH* exon 1 was present in all fibroid tissue. This is a novel and rare type of mutation in *FH* that has not been reported before in uterine fibroids and other *FH*-related diseases. Taken together (L39_F45 deletion in *MED12* and L28L missense in *FH* found in this patient), we suspect a potential involvement of these mutations that promote tumorigenesis through their distinct mechanisms.

In Patient Two, we found three missense mutations affecting codon G44D, G44R, G44S and a deletion mutation K42_G44 in *MED12* exon 2 in the medium and large size fibroid, respectively (Table 1 and Supplementary Figure S1). All of these changes were clustered around codon 44, which is the frequent hotspot for *MED12* mutation, and again, this N-terminal site (start of 100 amino acids, encoded by exon 1 and 2 in *MED12*) of *MED12* is essential for protein-protein interaction. Notably, one (sample ULM7.6) out of these four *MED12* mutation-positive fibroid also have missense mutation (M1L) in exon 1 of *FH*. In the case of this patient, it sets the foundation for further investigation to analyse the protein expression patterns in *FH* mutation-positive and mutation-negative uterine fibroids and relate to *MED12*. The outcome of this investigation will lead to the identification of protein expression patterns associated with *MED12* and *FH* as biomarkers for diagnosing uterine fibroids. For *FH*-negative mutation fibroids (in ULM7.1, ULM7.2, ULM7.3, ULM7.4 and ULM7.8) observed in this patient, the presence of other genetic mutations such as *MED12* are still active in the subset of these fibroids, and the *MED12* mutation alone can drive tumour formation (Table 1 and Figure 2). This evidence is in line with a mouse model study that the expression of missense mutant *Med12* can be the sole cause of uterine fibroids (23). Consequently, the impact of other driver genetic mutations, including *HMGA2* and *COL4A5-COL4A6,* on fibroid protein expression requires further investigation.

In Patient Three, we saw majority of the various size fibroids and ANM harbouring either missense and multiple mutations affecting codon R6P, L8P, A9P and V15L in *FH* exon 1 (Table 1). Again, this is the third patient in our study which reported mutation in the N-terminal of *FH* exon 1. This first exon of *FH* encodes the mitochondrial signal peptide of fumarase that targets the proteins to the mitochondria (24). It is tempting to speculate that the functional consequences of mutation in this *FH* region may potentially abolish mitochondrial targeting and import and subsequently lead to the loss of enzymatic activity. Ongoing and future research should explore this particular region exon 1 of *FH* and relate to the uterine fibroid. As opposed to the *FH* exon 1 mutation, we detected two small fibroids with a missense mutation, affecting codon 44 in exon 2 of *MED12*, and one of them carries the *FH* mutation (Figure 1 and Table 1).

Combining these three-patient case studies proves that *MED12* and *FH* mutations co-exist, possibly driving and competing for the same roles in tumorigenesis of uterine fibroid. From the immunopathological analysis carried out on our cohort, no distinct mitotic advantage was seen between fibroids with mutations in *MED12* only or *MED12 *+ *FH* genes. Alternatively, these two genes, *MED12* and *FH*, are also likely to act independently irrespective of *MED12* or *FH* pathway, as discussed by another group. Various germline *FH* mutations have been reported previously in women patients associated with HLRCC syndrome (25). Missense mutations appear to be more frequently detected mutations in *FH* than frameshift, nonsense and splice site in the HLRCC patients (26). This is, to our knowledge the first evidence and genetic annotation of fibroids in Australian women that harbor both somatic *MED12* and *FH* mutation.

## Data Availability

The original contributions presented in the study are included in the article/**Supplementary Material**, further inquiries can be directed to the corresponding author/s.
